# Immunotherapy of cancer without induction of autoimmunity – CD6 as a therapeutic target

**DOI:** 10.3389/fimmu.2026.1845422

**Published:** 2026-08-11

**Authors:** David A. Fox, Mikel Gurrea-Rubio

**Affiliations:** Division of Rheumatology and Program in Translational Immunology, University of Michigan, Ann Arbor, Michigan, MI, United States

**Keywords:** autoimmunity, cancer immunotherapy, CD6, CD8+ T cells, immune checkpoint inhibitors, NK cells, regulatory T cells

## Abstract

The CD6 cell surface glycoprotein is expressed by almost all T cells, a small subset of B cells and a substantial proportion of human natural killer (NK) cells. CD6 has multiple ligands and multiple functional epitopes. It is a component of the immunological synapse, and can positively or negatively influence signal transduction in T cells through complex interactions with multiple kinases and adapter molecules. Antibodies to CD6 are effective in the treatment of autoimmune diseases in experimental systems and in humans. The recent discovery that CD318 (CDCP1), a driver molecule in many cancers, is a ligand for CD6 has prompted investigation of CD6 as a possible new target for immunotherapy of cancer. A monoclonal antibody to CD6 rapidly internalizes CD6 and alters gene expression in CD8+ T cells and NK cells to augment the cytotoxicity of human lymphocytes to human cancer cells from a range of neoplasms. This review, using a question and answer format, describes recent work on anti-CD6 as a candidate immunotherapy for cancer as well as relevant advances in our understanding of other aspects of the biology of CD6, and explores the possibility that this approach could avoid the autoimmune complications that are encountered with checkpoint inhibitor treatment of cancer.

## What is the role of CD6 in the thymus?

The gradual increase in CD6 expression as developing thymocytes progress through their differentiation stages in the thymus closely parallels the gradual increase in expression of the CD3/TCR complex (1, 2). This suggests that CD6 may functionally interact with CD3/TCR during negative and positive selection. Moreover, CD6 expression on thymocytes directly correlates with the expression of the positive selection marker CD69 (1).

The action of CD6 in the thymus needs to be understood in the context of experiments that used *CD6^-/-^* mice in models of autoimmune disease, that showed striking clinical protection, and inhibition of the development of Th effector cells that could secrete cytokines such as gamma-interferon, IL-17 and IL-9. Similar results were obtained with the use of an anti-CD6 monoclonal antibody (mAb) in CD6-humanized mice (3–5). These results are consistent with the suggestion that CD6 enhances positive selection in the thymus. In the absence of CD6 mildly autoreactive thymocytes that had evaded negative selection would not subsequently undergo positive selection and clonal expansion, thus reducing the overall autoreactivity of the T cell repertoire.

One report proposes that thymic Treg generation is defective in *CD6^-/-^* mice, and that these mice had worse collagen-induced arthritis (CIA, a model of rheumatoid arthritis) compared to *CD6^+/+^* control mice (2). This contrasts with other reports which found that, as in EAE (experimental allergic encephalomyelitis, a model of multiple sclerosis) and uveitis, *CD6^-/-^* mice were protected from CIA (3–5). An important factor that might explain this discrepancy is the use of different strains of mice. The mice that were protected from CIA by CD6 deficiency were from the autoimmune-prone DBA strain, which is the optimal mouse strain for induction of robust, clinically obvious CIA, and for testing of interventions that could ameliorate CIA. The mice in which CIA was enhanced by CD6 deficiency were of the C57black strain, in which CIA was so mild that it was barely clinically detectable. It is also possible that different environmental conditions can influence the incidence and severity of CIA by distinct alterations of microbiomes.

## How does CD6 affect the response of T cells following activation through the T cell antigen receptor/CD3 (TCR/CD3) complex?

CD6 is not only a structural component of the immunological synapse, but also a functionally important regulator of differentiation pathways of effector T-cells involved in pathogenic CD4^+^ T-cell responses. The long cytoplasmic tail lacks intrinsic enzymatic activity but serves as a signaling scaffold through phosphorylation-dependent recruitment of adaptor proteins (6). Accordingly, CD6 has emerged as a key regulator of immune synapse organization and T-cell receptor (TCR)-driven responses, with context-dependent effects on lymphocyte activation (6–9). However, expression and function of CD6 does not require co-expression of CD3, since approximately 50% of human (but not mouse) natural killer (NK) cells are CD6+, but CD3-, and the anti-CD6 mAb UMCD6 profoundly alters the gene expression program of these NK cells leading to activation and heightened cytotoxicity against cancer cells (10), similar to the effects of UMCD6 on CD3+CD8+ T cells (11, 12).

In CD4+ cells CD6 signals by acting as what has been termed a transmembrane “rheostat signalosome” that balances T-cell activation, primarily through its long cytoplasmic tail, which interacts with both activating and inhibiting kinases and adaptors (8, 13). It operates via two main mechanisms: a ligand-dependent pathway where binding to its partner ALCAM (CD166) stabilizes the immunological synapse, and a ligand-independent (tonic) inhibitory mechanism that tunes the TCR signaling threshold. CD6 localization to the immunologic synapse does not require engagement of CD166/ALCAM a CD6 ligand) or activation of CD3/TCR by antigen, but can be augmented by those processes (8, 13, 14). Components of the CD6 signalosome include linker molecules (SLP76, TSad, GADS and VAV1) kinases that can augment the activation signal (Lck, Fyn, ZAP-70 and Ltk) and adapters and other inhibitors that can lower the signal and/or target components of the synapse for degradation (SHP-1, RasGAP, CBL-B and UBASH3A) (6–10, 13, 14). Thus, the consequences of engagement of CD6 by external ligands will depend on relative availability of the various proteins that associate with the long cytoplasmic tail of CD6. A downstream event that can occur when the CD6 signalsome is reinforcing an activating signal is the activation of protein kinase C theta, although, unlike CD28, CD6 does not bind directly to PKC theta (15). Importantly, apparently divergent observations regarding CD6 function may reflect major differences in the mode and strength of T-cell activation being studied. Responses induced by supraphysiologic anti-CD3/anti-CD28 stimulation can differ substantially from those observed in more physiologic systems that depend on stable T cell–APC interactions and immune synapse organization. In this context, the adhesive and synapse-organizing properties of CD6 are likely to become particularly important. These considerations may help explain why distinct anti-CD6 antibodies, CD6-deficient systems, or different experimental settings can produce divergent biological outcomes depending on epitope specificity, receptor internalization, ligand interference, cellular subset, or inflammatory microenvironment.

## How might CD6 targeting affect regulatory T cells and the risk of immune-related adverse events?

CD6 has most often been studied in the context of conventional effector T-cell activation, but its role in regulatory T-cell biology is also relevant to the prediction that CD6-targeted cancer immunotherapy may have a lower risk to induce immune-related adverse events than checkpoint blockade. In effector CD4+ T cells, CD6 can promote immune synapse stability and support Th1/Th17-type inflammatory responses under appropriate conditions. This is consistent with the protection from experimental autoimmune encephalomyelitis, uveitis, and collagen-induced arthritis observed after genetic deletion or antibody-mediated targeting of CD6 in several model systems (3–5). In these settings, anti-CD6 therapy appears to reduce pathogenic effector T-cell differentiation or function without causing profound lymphocyte depletion.

The role of CD6 in regulatory T cells is likely more complex and remains incompletely defined. One study reported impaired thymic regulatory T-cell generation in CD6-deficient mice, accompanied by worsened collagen-induced arthritis in a C57BL/6 background (2), whereas other studies found protection from autoimmune disease in CD6-deficient or anti-CD6-treated animals (3–5). These apparently divergent observations suggest that CD6 may influence Treg development, peripheral Treg fitness, or suppressive function in a manner that depends on genetic background, inflammatory context, microbiome, antigenic stimulus, and the particular experimental model. Thus, CD6 may have distinct effects on thymic Treg selection, conventional effector T-cell activation, and peripheral immune homeostasis.

**Figure 1:**
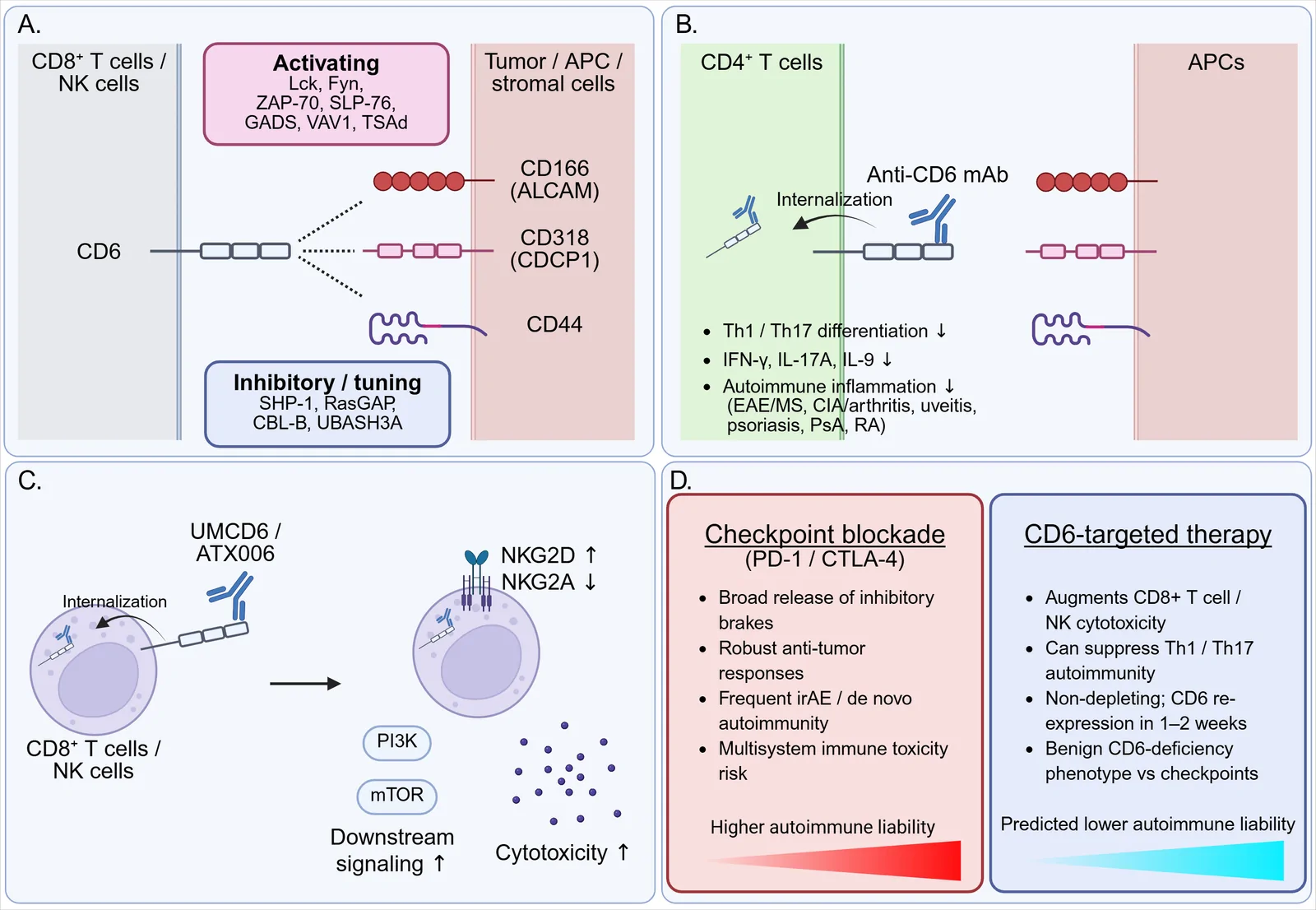
CD6-targeted immunotherapy may dissociate anti-tumor efficacy from autoimmune toxicity. **(A)** CD6 is a multi-ligand immunoregulatory receptor expressed on T cells and a subset of human NK cells that binds CD166/ALCAM, CD318/CDCP1, and CD44, and functions as a context-dependent signaling scaffold at the immune synapse. **(B)** In autoimmune settings, anti-CD6 antibodies induce rapid capping/internalization of CD6 and suppress pathogenic Th1/Th17-type CD4+ T-cell responses. **(C)** In cancer, the anti-CD6 monoclonal antibody UMCD6 (humanized form ATX006) reprograms CD8+ T cells and NK cells toward enhanced cytotoxicity, including increased activating programs (e.g., NKG2D-associated) and reduced inhibitory programs (e.g., NKG2A-associated), resulting in increased tumor cell killing. **(D)** In contrast to PD-1/CTLA-4 checkpoint blockade, which often causes immune-related adverse events, CD6-targeted therapy may provide anti-tumor activity with lower autoimmune liability, consistent with the relatively benign phenotype of CD6 deficiency. The non-depleting properties of anti-CD6 antibodies, and recovery of surface CD6 expression within 1–2 weeks after internalization could further enhance the safety of this approach.

This issue is especially important in cancer immunotherapy. In contrast to genetic deficiency of PD-1, PD-L1, or CTLA-4, which is associated with severe spontaneous autoimmunity, CD6 deficiency does not produce a comparable multisystem autoimmune phenotype (3–5). In addition, clinical experience with anti-CD6 antibodies in autoimmune diseases has not revealed a strong signal for induction of new autoimmune syndromes. These observations support the hypothesis that CD6-directed cancer immunotherapy may enhance cytotoxic lymphocyte function without broadly disabling dominant pathways of peripheral tolerance. Future clinical trials should therefore include careful assessment of Treg number, phenotype, suppressive function, tissue localization, and stability, as well as longitudinal monitoring for immune-related adverse events. The recently described CD318+ CD8+ regulatory T-cell population further highlights the need to understand how the CD6-CD318 axis influences both cytotoxic and regulatory lymphocyte subsets (16) (**Figure 1**).

## Does CD6 have more than one ligand?

Yes - it has several- both mammalian and microbial (**Table 1**). The first mammalian ligand of CD6 to be discovered was CD166/ALCAM (Activated Cell Leucocyte Adhesion Molecule) (17), which is believed to function as a cell adhesion molecule that stabilizes interactions between CD6+ cells and the many cell types that express CD166.

**Table 1:** Ligands of CD6.

Ligand category	Ligand name	Alternative name	Relevance	Key references
Mammalian	CD166	ALCAM; activated leukocyte-cell adhesion molecule	First identified mammalian CD6 ligand; adhesion molecule that stabilizes CD6-dependent cell-cell interactions and contributes to immune synapse organization	Bowen et al. (17); Goncalves et al. (8)
Mammalian	CD318	CDCP1; TRASK	Second mammalian CD6 ligand; expressed by several epithelial, stromal, inflammatory, and malignant cells, implicated in cancer biology and lymphocyte interactions with tumor cells	Joo et al. (18); Saifullah et al. (19); Enyindah-Asonye et al. (20); Ruth et al. (11)
Mammalian	CD44	Hyaluronic acid receptor	CD6 ligand that can participate with CD166 and CD318 in multimolecular receptor complexes and cytoskeletal remodeling	Borjini et al. (21)
Mammalian/Soluble	Soluble CD318	sCD318	Detected in inflammatory biological fluids; has chemoattractant activity for T cells and NK cells, although the mechanism remains incompletely defined	Saifullah et al. (19); Enyindah-Asonye et al. (20)
Bacterial	Lipoteichoic acid	LTA	Gram-positive bacterial component recognized in the context of CD6 scavenger-like pattern recognition	Sarukhan et al. (22); Català et al. (23)
Bacterial	Peptidoglycan	PGN	Gram-positive bacterial cell-wall component implicated in CD6-mediated recognition of bacterial products	Sarukhan et al. (22); Català et al. (23)
Bacterial	Lipopolysaccharide	LPS	Gram-negative bacterial component; relevant to studies of CD6 in innate immune responses and sepsis	Sarukhan et al. (22); Català et al. (23)
Viral	HIV-1 gp120 V3 loop	V3 loop of gp120	Reported microbial ligand-like interaction involving CD6; biological significance requires further clarification	Sarukhan et al. (22)
Other microbial	Echinococcus-derived ligand(s)	Not fully defined	Proposed interaction; requires further molecular characterization	Sarukhan et al. (22)
Other microbial	Fungal ligand(s)	Not fully defined	Proposed interaction; requires further molecular characterization.	Sarukhan et al. (22)

CD6 binds several mammalian ligands and can also recognize selected microbial products, consistent with its classification as a scavenger receptor-like lymphocyte surface molecule. The functional outcome of CD6 engagement likely depends on ligand identity, cellular context, receptor isoform, epitope engagement, and the composition of the CD6-associated signalosome.

Shortly after the discovery of CD166 as a ligand of CD6, indirect evidence began to appear that suggested the existence of a second ligand. In binding assays involving the interaction of T cells with either keratinocytes (KC) or synovial fibroblasts (FLS), binding was increased by gamma-interferon, yet CD166 expression was not altered. Moreover, a CD6 fusion protein specifically immunoprecipitated both CD166 and a protein of higher apparent molecular mass than CD166 from gamma interferon-treated KC or FLS (18). A mAb was raised against this protein (19), that was ultimately used to purify the non-CD166 CD6 ligand, sequence of which revealed it to be CD318, more commonly known as CDCP1 or TRASK (20). Multiple approaches were used to confirm that CD318 was, indeed, a second ligand of CD6 (20).

More recently, CD44, a cell surface receptor for hyaluronic acid, was found to be a third ligand of CD6 (21). These three ligands can function in a coordinated manner and assemble into a multimolecular complex on retinal epithelial cells (21), and likely other cell types. On gamma-interferon-treated FLS, blocking antibodies against both CD166 and CD318 are required to interfere with adhesion of T cells (20).

The microbial ligands of CD6 include important stimuli of innate immune defenses against bacteria (22). Fortunately, other receptors on cells other than lymphocytes also are engaged by these bacterial components (such as toll like receptors and CD14 in and on monocytes). Nevertheless, *CD6^-^*^/-^ mice are defective in coping with bacterial sepsis (23). Thus far, use of an anti-CD6 mAb itolizumab, in the treatment of psoriasis and psoriatic arthritis in Cuba and India, has not shown a signal for infection as a toxicity (24, 25). This may reflect redundancy in innate host defenses against bacteria, because many cell types express pattern-recognition receptors capable of recognizing multiple bacterial ligands. In the absence of CD6, other receptors could hypothetically compensate for at least some of the scavenger receptor functions of CD6.

## Which autoimmune diseases could be selected for treatment with anti-CD6?

The good efficacy and safety track record of itolizumab in treating psoriasis, psoriatic arthritis and RA (24–26) is likely to prompt consideration of anti-CD6 mAbs for those and other autoimmune diseases Conditions that are dependent on Th1 and/or Th17 cells are the most logical candidates, in view of very robust results using anti-CD6 in model systems in CD6-humanized mice.

Anti-CD6 antibodies can rapidly cap and internalize CD6, which could explain the lack of severe lymphopenia in anti-CD6 treated mice or humans, if internalization occurs faster than the deployment of mechanisms for elimination of anti-CD6-coated lymphocytes.

Logical treatment targets could include rheumatoid arthritis, multiple sclerosis, sterile uveitis, type 1 diabetes, Sjogren’s disease and many other conditions. Autoantibody-mediated diseases can potentially be included on this list as long as the autoantibody response is known to be T cell-dependent.

The ability of anti-CD6 to inhibit release of Th1 and Th17 cytokines also led to a reportedly successful trial in cytokine storm complicating SARS-Cov2 infection, raising the possibility of considering trials of anti-CD6 in other autoinflammatory syndromes (27). Known associations between *CD6* polymorphisms and various diseases could also be a factor for prioritization of clinical trials. For example, *CD6* is a gene locus strongly associated with multiple sclerosis, with the disease associated allele creating a stronger CD6 promotor (28).

## How does anti-CD6 induce immune cells to kill cancer cells, by mechanisms distinct from checkpoint inhibition?

All three mammalian CD6 ligands are widely expressed on cancer cell lines. Of special interest are cancers, such as lung adenocarcinoma in which high expression of CD166 has an association with survival of the cancer patient (longer survival) that is robustly opposite to the effect of high expression of CD318 (shorter survival) (11). These results suggest that different CD6 ligands can generate different outcomes of signaling through CD6, with profound clinical consequences.

Preclinical studies have begun to address the potential for use in cancer immunotherapy of an anti-CD6 mAb termed UMCD6 (11, 12). (The humanized form of this mouse mAb is termed ATX006.) UMCD6 recognizes an epitope of CD6 that is unique compared to regions of CD6 recognized by other available anti-CD6 mAbs, consistent with data about multiple functionally and structurally distinct epitopes of CD6 that was generated more than 30 years ago, when this mAb was first described (29, 30). UMCD6 also has exceptionally high affinity and avidity for CD6 (30), which may account for the rapid and complete capping and internalization of CD6 that occurs with this mAb.

The internalization phenomenon has several implications, including the following:

1. Following internalization, none of the extracellular ligands of CD6 will be able to bind to CD6 until it is re-expressed on the cell surface (1–2 weeks later);
2. Associations of CD6 with other T cell surface molecules will be disrupted by rapid internalization of CD6;
3. An antibody-drug conjugate based on ATX006 would have potential as a treatment for CD6+ lymphoid neoplasms (31).

Other than the special case of CD6+ malignancies, use of anti-CD6 for treatment of cancer requires that the anti-CD6 mAb activates cytotoxic lymphocytes to kill the cancer cells. Extensive experimentation *in vitro* using multiple types of cancer cell lines and *in vivo* using immunodeficient mice that can accept xenografts of human cancers (breast and prostate) yield the following results and conclusions concerning UMCD6 (11, 12):

1. UMCD6 acts directly on CD8+ T cells, NK cells and NK T cells to augment killing capacity against almost all human cancer cell lines.
2. This functional change reflects extensive change in the pattern of gene expression in cytotoxic lymphocytes. Activating receptors such as NKG2D and downstream components of their signaling pathways are upregulated, while inhibitory receptors such as NKG2A are down-regulated.
3. Cancer cell killing by lymphocytes stimulated by UMCD6 is typically greater than or equal to effects of anti-PD1.
4. In xenograft mouse models, engrafted mice with visible tumors are treated by injecting 10–15 x 10^6^ human lymphocytes i.v. followed by 0.1 mg UMCD6 i.p. These mice have longer survival than controls, but are not cured, likely due to gradual loss of the infused human lymphocytes.
5. By day 2 of treatment, growth of xenografted cancers is halted. By day 4 tumor shrinkage is generally evident, accompanied by infiltration of CD8+ T cells and NK cells in approximately equal numbers.
6. Allogeneic effects due to MHC mismatches between cancer cells and infused human lymphocytes are not a sufficient explanation for these results. In an autologous system (cancer organoids) UMCD6 induces prompt organoid destruction as long as autologous lymphocytes are present within the organoid (12).

These observations favor classification of UMCD6 as a selective lymphocyte engager, not a checkpoint inhibitor. This distinction is reinforced by the absence of spontaneous autoimmunity in mice that are genetically deficient in CD6 or its ligands, and the protection from induction of autoimmunity in all models of human autoimmune disease thus far tested. In contrast, genetic absence of *PD1, PDL1* or *CTLA4* engenders an overwhelming multisystem autoimmune diathesis.

## What other aspects of CD6 require further study?

Without implying that our understanding of any aspect of CD6 biology is complete, a few questions of special interest or importance can be posed.

1. What is the functional importance of the existence of multiple isoforms of CD6?,
2. How is expression of CD6 regulated?
3. Does engagement of CD6 by a CD6 ligand lead to conformational changes in the cytoplasmic tail, distinct for each ligand, that define pathways for downstream signaling events?
4. Can anti-CD6 prevent or treat transplant rejection?
5. Will anti-CD6 be useful in the management of autoinflammatory conditions?
6. Soluble/shed CD318 (sCD318) is measurable in biological fluids such as synovial fluid from patients with inflammatory arthritis. sCD318 has chemoattractant properties for T cells and NK cells, but CD6 lacks the structural elements of chemoattractant receptors, which are G-protein coupled receptors (GPCRs) with multiple transmembrane domains. Is there a CD6-associated GPCR that is a coreceptor for sCD318?
7. Can the chemoattractant properties of sCD318 be exploited in cancer immunotherapy?
8. Can tumors acquire resistance to CD6-directed immunotherapy by altering expression of CD6 ligands, including reciprocal changes in CD166/ALCAM, CD318/CDCP1, or CD44?
9. Does sustained anti-CD6 therapy select for tumor microenvironments enriched in suppressive myeloid cells, regulatory T cells, soluble inhibitory mediators, or altered extracellular matrix components that reduce lymphocyte infiltration or cytotoxicity?
10. After prolonged CD6-targeted therapy, do cytotoxic lymphocytes compensate by upregulating inhibitory receptors or exhaustion-associated pathways such as PD-1, TIM-3, LAG-3, TIGIT, NKG2A, KIR-family receptors, or other negative regulators?

## Discussion

Although immunotherapy with checkpoint inhibitors has revolutionized the treatment of many forms of cancer, significant limitations exist due to autoimmune toxicities and insufficient clinical responses. These two problems intersect when use of an effective dose/duration of treatment is not possible due to a very high likelihood of life-threatening complications. These obstacles were readily predictable from the phenotype of knockout mice.

Although CD6 biology is complex and context dependent, several features nevertheless make CD6 an attractive target for cancer immunotherapy. The phenotype of *CD6^-/-^* mice is reassuringly benign. A defect in host defense against bacterial sepsis has been elicited in these mice but has not yet been paralleled by observations in humans treated long term with anti-CD6 for various autoimmune diseases in India, a location with notably high prevalence of many chronic infectious diseases.

The ability of anti-CD6 to treat autoimmunity underscores the inference that autoimmune toxicities would not be likely if CD6 became a target for cancer treatment. It is appreciated that the pathogenesis of checkpoint inhibitor autoimmune complications may differ from previously characterized autoimmune diseases and not always be Th1/Th17 driven. We need to understand much more about the role of CD6 in the function of regulatory T cells, and about a recently-described subset of CD8+ T cells that are CD318+ and have regulatory properties (16). Nevertheless, putting together the currently available clues, it appears that induction of new autoimmune diseases would become the exception rather than the rule, with CD6-targeted cancer immunotherapy.

Important limitations of the current preclinical evidence should also be acknowledged. The available cancer studies of UMCD6 have largely used *in vitro* systems, human tumor organoids, and xenograft models in immunodeficient mice supplemented with human lymphocytes. These systems are valuable for demonstrating direct effects on human CD8+ T cells and NK cells, but they cannot fully reproduce the complexity of an intact human tumor microenvironment or the long-term immunologic consequences of repeated dosing. Potential mechanisms of therapeutic resistance include tumor-cell adaptation through altered expression of CD6 ligands, particularly changes in the relative abundance of CD166/ALCAM, CD318/CDCP1, and CD44; shedding of soluble ligands that could modify immune-cell trafficking or receptor engagement; impaired lymphocyte infiltration; and emergence of suppressive stromal or myeloid programs. In addition, sustained activation of cytotoxic lymphocytes could theoretically be followed by compensatory upregulation of inhibitory receptors or exhaustion-associated pathways, such as PD-1, TIM-3, LAG-3, TIGIT, NKG2A, KIR-family receptors, or other negative regulators. These possibilities argue for incorporating serial tumor biopsies, ligand-expression profiling, single-cell transcriptomic and proteomic analyses, and longitudinal immune monitoring into early-phase clinical trials of ATX006 or related anti-CD6 strategies.

Finally, the non-depleting properties of anti-CD6 mAbs, with recovery of CD6 cell surface expression in 1–2 weeks could mean that recovery from an unexpected toxicity would be rapidly facilitated simply by a temporary interruption in the dosing schedule.

Clinical trials of anti-CD6 in a variety of cancers will provide an opportunity to test these hypotheses, ask some of the questions and determine the accuracy of the predictions that are made in this review.

## Disclosure

DF is a cofounder of and holder of equity in Abcon Therapeutics, a company that is focused on developing anti-CD6 as a treatment for cancer and autoimmune diseases.
